# Circulating Biomarkers in Muscular Dystrophies: Disease and Therapy Monitoring

**DOI:** 10.1016/j.omtm.2020.05.017

**Published:** 2020-05-22

**Authors:** Andrie Koutsoulidou, Leonidas A. Phylactou

**Affiliations:** 1 Department of Molecular Genetics, Function & Therapy, The Cyprus Institute of Neurology and Genetics, PO Box 23462, 1683 Nicosia, Cyprus; 2 Cyprus School of Molecular Medicine, The Cyprus Institute of Neurology and Genetics, PO Box 23462, 1683 Nicosia, Cyprus

## Abstract

Muscular dystrophies are a group of inherited disorders that primarily affect the muscle tissues. Across the muscular dystrophies, symptoms commonly compromise the quality of life in all areas of functioning. It is well noted that muscular dystrophies need reliable and measurable biomarkers that will monitor the progress of the disease and evaluate the potential therapeutic approaches. In this review, we analyze the current findings regarding the development of blood-based circulating biomarkers for different types of muscular dystrophies. We emphasize those muscular dystrophies that gained particular interest for the development of biomarkers, including Duchenne muscular dystrophy, Becker muscular dystrophy, myotonic dystrophy types 1 and 2, Ullrich congenital muscular dystrophy, congenital muscular dystrophy type 1A, Facioscapulohumeral muscular dystrophy, and limb-girdle muscular dystrophy types 2A, 2B, 2C, and 2D, recently renamed as limb-girdle muscular dystrophy R1 calpain3-related, R2 dysferlin-related, R5 γ-sarcoglycan-related, and R3 α-sarcoglycan-related. This review highlights the up-to-date progress in the development of biomarkers at the level of proteins, lipids, and metabolites, as well as microRNAs (miRNAs) that currently are the main potential biomarker candidates in muscular dystrophies.

## Graphical Abstract

**Figure:**
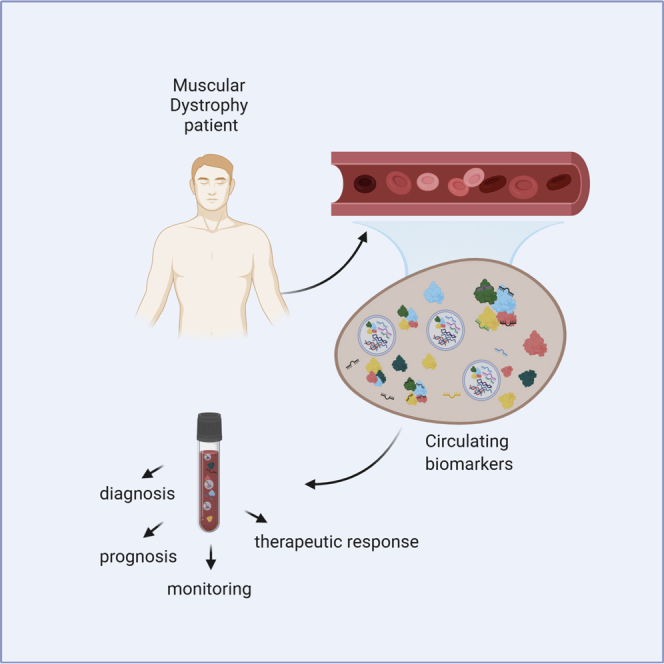

## Teaser

Muscular dystrophies are a family of inherited disorders. Apart from the need to develop therapeutic drugs, an increasing interest exists for the development of biomarkers. Koutsoulidou and Phylactou review the current findings related to the circulating biomarkers and their potential use as prognostic, monitoring, or pharmacodynamic biomarkers for muscular dystrophies.

## Main Text

Muscular dystrophies are a heterogeneous group of inherited genetic muscle disorders affecting both children and adults. Currently, more than 50 distinct types of muscular dystrophies have been identified that are inherited in an autosomal dominant, autosomal recessive, or X-linked fashion. Very rarely, muscular dystrophy can occur sporadically. They all share similar clinical and pathological characteristics, including skeletal muscle weakness, wasting, and degeneration.^1^ Different mutations in a variety of genes are associated with the phenotype of each muscular dystrophy.^1^ The pathology of each disorder differs, reflecting various clinical characteristics, including the groups of primarily affected muscles, the degree of weakness, the time of onset, and the rate of progression. For most of the muscular dystrophies, diagnosis is well established; however, additional disease parameters, such as the progression of the disease and the response to therapeutic interventions, remain uncharacterized. This has resulted in a pressing need for the development of reliable and measurable biomarkers for both the monitoring of the disorder and the response to therapeutic approaches that concern this family of disorders.

**Figure 1:**
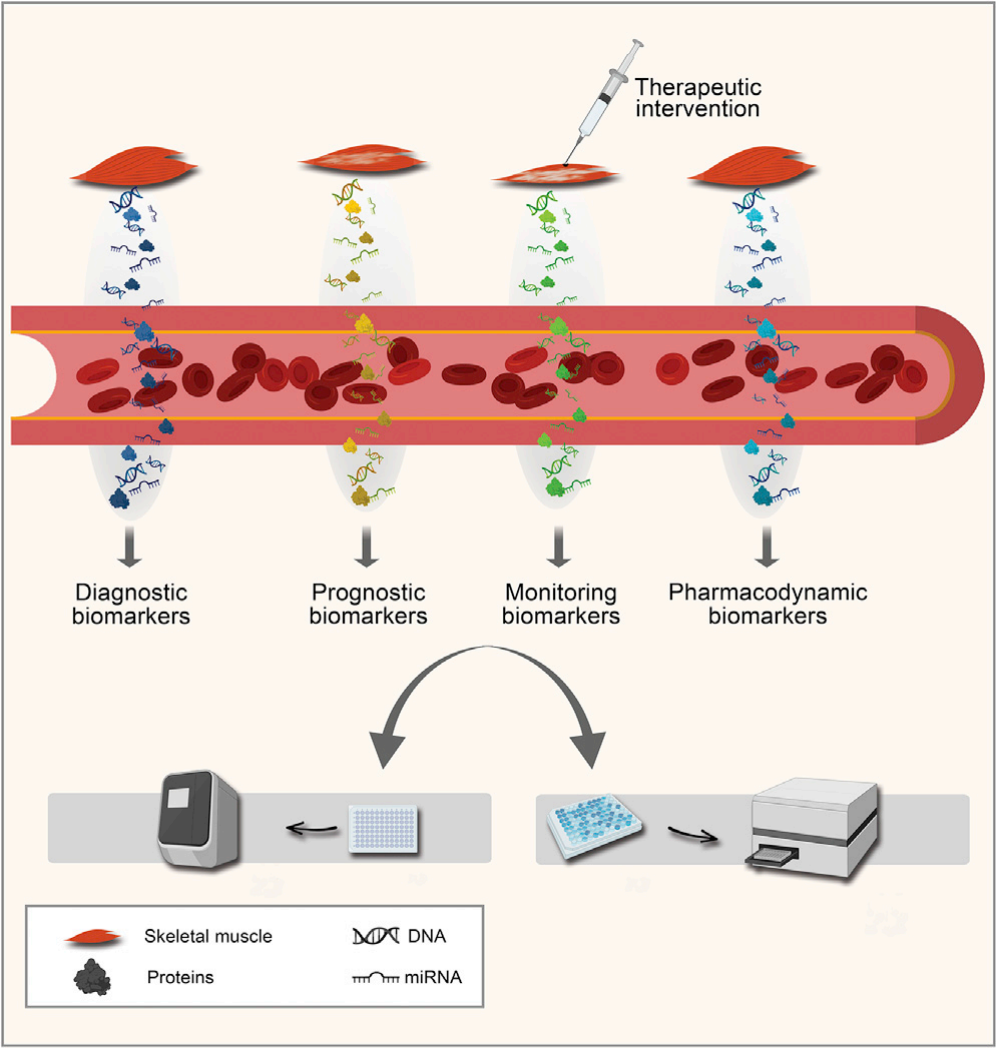
Different Types of Biomarkers Present in Blood Biomarkers are measurable indicators that reflect normal biological processes, pathogenic conditions, or responses to therapeutic interventions. In blood, three main types of circulating biomarker exist: DNA, RNA, and proteins/metabolites. Depending on the nature of the biomarker, different detection techniques are employed. Different categories of biomarkers have been defined and recorded, including diagnostic, monitoring, prognostic, and pharmacodynamic/response biomarkers. Diagnostic biomarkers are used to identify individuals with a disease or condition of interest or to define a subset of the disease. Prognostic biomarkers are used to detect the possibility of a clinical symptom, disease recurrence, or disease progression. This type of biomarkers will provide added value to the diagnosed patients for the early detection of clinical symptoms. Monitoring biomarkers are used to regularly screen the patients for a change in the degree or extent of disease. Regarding muscular dystrophies, monitoring biomarkers are currently of urgent need in order to evaluate the progress of the disease. Pharmacodynamic/response biomarkers are used to evaluate the response of the patient to a therapeutic intervention. Considering that currently there is an intense interest to develop therapeutic approaches for muscular dystrophies, the need to develop pharmacodynamic/response biomarkers for muscular dystrophies is rapidly increasing.

Biomarkers are measurable indicators of normal or pathogenic conditions or pharmacological responses to a therapeutic intervention. Different types of biomarkers were reported and classified based on their use (Figure 1).^2^ For muscular dystrophies emphasis has been given on the development of circulating biomarkers for monitoring the progress of the disorder (monitoring biomarkers) and the response to therapeutic approaches (pharmacodynamic biomarkers). The evaluation of the progress of the disorder by measuring non-invasive biomarkers will provide to the clinicians and patients an invaluable tool for patient care. Herein, we review the current knowledge on the presence of potential biomarkers in the blood of patients with various types of muscular dystrophies. Despite that the family of muscular dystrophies consists of many disorders, the main research emphasis regarding the development of biomarkers has been placed on Duchenne muscular dystrophy (DMD), probably due to the higher frequency and the severity of the disease of the young affected boys. Work has also been performed on some of the other muscular dystrophies, including Becker muscular dystrophy (BMD), myotonic dystrophy types 1 and 2 (DM1 and DM2), Ullrich congenital muscular dystrophy (UCMD), congenital muscular dystrophy type 1A (MDC1A), facioscapulohumeral muscular dystrophy (FSHD), and limb-girdle muscular dystrophy types 2A, 2B, 2C, and 2D (LGMD2A, LGMD2B, LGMD2C, and LGMD2C), recently named as limb-girdle muscular dystrophy R1 calpain3-related, R2 dysferlin-related, R5 γ-sarcoglycan-related, and R3 α-sarcoglycan-related.^3^

### Strong Interest for Developing Biomarkers for DMD

DMD is an X-linked disorder caused by mutation in *DMD* gene abolishing the expression of dystrophin protein. DMD is a severe progressive muscular dystrophy primarily among children. It is clinically characterized by progressive muscle necrosis and wasting, leading to loss of ambulation by 8–12 years of age and premature death by early adulthood due to cardiorespiratory failure.^4^ BMD is a milder form of DMD caused by mutation on the same gene that encodes a shortened and partially functional protein. BMD manifests later in life with an average onset of 12 years, delayed loss of ambulation until the third decade of life, and in some cases without any loss of ambulation and variable onset of cardiac involvement.^4^ In recent years, several studies were performed aiming to identify molecular biomarkers for monitoring the disease since the methods currently being used have been determined to have many drawbacks.^5^^,^^6^ Emphasis has been also given to develop pharmacodynamic biomarkers to evaluate the efficiency of therapeutic approaches. Research was mainly focused on the presence of circulating microRNAs (miRNAs) and proteins in the blood of DMD patients.^7^, ^8^, ^9^ A series of potential biomarkers were reported in both DMD patients and DMD animal models, including *mdx* mice, dystrophin/utrophin double-knockout (dKO) mice, golden retriever muscular dystrophy (GRMD), and canine X-linked muscular dystrophy in Japan (CXMD_J_) dogs.^10^

### Detecting miRNAs in Blood of DMD and BMD Patients

miRNAs are small endogenous regulatory non-coding RNA molecules that play an important role in the regulation of numerous biological processes. Recently, significant amounts of miRNAs were detected in extracellular body fluids, such as blood serum and plasma, and proposed as potential non-invasive biomarkers for various diseases.^11^

Many miRNAs are expressed in a tissue-specific or developmental stage-specific manner. Four miRNAs specifically expressed in muscle tissue, that is, miR-1, miR-133a, miR-133b, and miR-206, known as myomiRs, were extensively studied for their expression and function in muscle.^12^, ^13^, ^14^ The four myomiRs were found elevated in blood of DMD patients and DMD animal models, including *mdx* mice, CXMD_J_ dogs, and GRMD dogs, compared to controls.^15^, ^16^, ^17^, ^18^, ^19^, ^20^, ^21^ Notably, the levels of the myomiRs were correlated with the disease severity and clinical assessments of DMD patients, implying that they can serve as monitoring biomarkers for the disorder.^15^^,^^18^^,^^22^ Enrichment of the myomiRs was also reported in BMD individuals compared to controls at a lower level compared to DMD patients.^18^ The increase in the severity of DMD disorder in relation to BMD could possibly explain the difference observed in myomiR levels.

In recent years, there has been an intense interest to develop therapeutic interventions for DMD. The absence of measurable pharmacodynamic biomarkers revealed a problem that urgently needs to be solved. One of the most promising methods to tackle DMD and BMD involves the use of antisense oligonucleotides in *mdx* mice.^23^ The *mdx* mouse model has been widely used for the development of therapeutic approaches for DMD. *mdx* mice are developed through a point mutation in exon 23, resulting in premature termination of dystrophin expression. The concept behind exon skipping approaches in *mdx* mice is to splice out exon 23 from precursor (pre-)mRNA and generate a translatable transcript from the mutant *DMD* gene. Comparison of different exon skipping tools revealed a dose-dependent correlation between dystrophin rescued levels and the restoration of myomiR levels.^17^^,^^19^ These results show a promising potential use of these miRNAs as pharmacodynamic biomarkers in DMD.

The serum levels of myomiRs were also investigated in DMD patients who participated in two clinical trials testing the exon skipping therapy intervention by using the phosphorodiamidate morpholino oligomer (PMO) known as eteplirsen.^22^^,^^24^ Eteplirsen is a PMO that targets exon 51 of the *DMD* gene pre-mRNA transcript so that exon 51 is excluded, or skipped, from the mature posttranslational spliced mRNA. As a result, the disrupted reading frame of DMD is restored, and a functional, but truncated, dystrophin protein is produced that converts the severe DMD phenotype to a milder BMD phenotype.^24^ None of the four myomiRs showed a significant difference in pre-treated and eteplirsen post-treated samples.^22^ The small number of patient samples in combination with the high variability of the myomiR levels in individual patients may explain the lack of a statistically significant difference between the pre-treated and post-treated samples.^22^ Additionally, the short duration of the study (12 weeks) may was not sufficient to evaluate the therapeutic response at the circulating miRNA levels.^22^ Experiments performed in *mdx* mice showed that a longer period of time (50 weeks) is required to increase dystrophin restoration, thus probably explaining the absence of a significant difference in myomiR levels in 12 weeks of treatment.^22^^,^^25^ A longitudinal study is required to evaluate the use of the myomiRs as pharmacodynamic biomarkers in DMD patients.

Additional miRNAs were sporadically identified to be differentially expressed in DMD. miR-22, miR-30a, miR-193b, miR-378, miR-128, miR-149, miR-378b, and miR-483 were found to be increased in *mdx* mice, although at a lower extent, compared to myomiRs.^19^^,^^26^ The elevated levels of miR-483, which was the most differentially expressed miRNA in *mdx* serum, were verified in DMD patients’ serum.^26^ Treatment of *mdx* mice with an antisense oligonucleotide restored the levels of these miRNAs toward wild-type levels.^19^^,^^26^ Additionally, miR-30c and miR-181a, which are highly expressed in muscle tissue, were reported as reliable serum-based diagnostic biomarkers for DMD, and miR-30c was found to correlate with the severity of the disease.^27^^,^^28^ These two miRNAs were also found to be elevated in BMD patients at a very similar magnitude as in DMD patients.^27^^,^^28^ A novel miR-188 was recently found to be elevated in the serum of the muscular dystrophy dog model, CXMD_J_, compared to normal dogs.^29^ A deeper investigation of miR-188 function showed that miR-188 is involved in muscle differentiation, and the serum levels reflect skeletal muscle regeneration.^29^

Although of high importance, very little information exists regarding the reasons underlying the stability of circulating miRNAs in DMD patients. Several studies reported different populations of circulating miRNAs. It is currently known that circulating miRNAs are released encapsulated into membrane vesicles, including microvesicles, exosomes, and apoptotic bodies, or bound to protein complexes.^30^^,^^31^ Primarily, it was reported that only a minority of serum myomiRs are found in extracellular vesicles, such as exosomes, whereas most are associated with protein/lipoprotein complexes.^19^ In an independent study, significant amounts of the myomiRs were found encapsulated within exosomes in serum of DMD patients.^32^ The use of different exosome isolation techniques by these studies may lead to the controversial reported results due to the variable efficiencies of the methods, the possible adherence of the exosomes to the filters, or the contamination of isolated exosomes with non-exosomal particles such as proteins.^33^

### Circulating Proteins as DMD Biomarkers

The serum and plasma proteome is considered a promising source of non-invasive biomarkers; however, identification of protein biomarkers has been limited due to its massive complexity.^34^ Interesting reports were published showing encouraging results regarding the potential use of proteins as blood-based biomarkers for DMD. These include muscle injury proteins, extracellular matrix remodeling proteins, muscle degeneration/regeneration-associated proteins, and inflammation/immune-related proteins. Plasma and serum of DMD patients were analyzed, facilitating either targeted approaches or high-throughput proteomics techniques.^8^^,^^35^, ^36^, ^37^ Two proteins involved in DMD pathogenesis, matrix metallopeptidase 9 (MMP-9) and tissue inhibitors of metalloproteinase-1 (TIMP-1), were found increased in the blood of *mdx* mice and DMD patients, compared to controls.^38^, ^39^, ^40^ Notably, MMP-9 levels were significantly higher in older, non-ambulant patients compared to the younger, ambulant patients, suggesting an inversely proportional relationship between MMP-9 circulating levels and disease progression.^38^, ^39^, ^40^ Treatment with the exon skipping 2′-*O*-methyl-phosphorothioate antisense oligonucleotide drug drisapersen did not affect MMP-9 serum levels, suggesting its potential use as only a monitoring biomarker.^40^ In addition to MMP-9, a large number of proteins that are significantly affected by disease progression were identified through longitudinal analysis of DMD patients.^36^ Fibronectin glycoprotein is one of these proteins suggested to be a promising biomarker for the progression of the disease with high disease specificity since its levels in BMD were comparable to control levels.^41^ Myostatin (GDF-8) and its antagonist follistatin (FSTN) were found altered in DMD patients and differentiated between BMD and LGMD patients.^39^ Four proteins that are abundant in skeletal muscle tissue, that is, myofibrillar proteins skeletal troponin I (sTnI), myosin light chain 3 (MYL3), fatty acid binding protein 3 (FABP3), and creatine kinase muscle-type (CKM), were determined to be significantly elevated in serum of both DMD and BMD patients and correlated with certain clinical endpoints in DMD patients, such as forced vital capacity.^42^^,^^43^ The serum levels of these proteins were also elevated in *mdx* mice and GRMD.^42^ CKM is an enzyme that leaks out of damaged muscle and can be used as an indicator of muscular dystrophy or inflammation without providing information regarding the exact form of the disorder. In addition to muscle function proteins, proteins that are involved in energy metabolism were determined to be altered in DMD and/or BMD patients.^43^ MYL3, carbonic anhydrase III (CA3), mitochondrial malate dehydrogenase 2 (MDH2), and electron transfer flavoprotein A (ETFA) were significantly different between DMD patients and both controls and female carriers.^43^ Further analysis of the circulating profiles of the identified proteins revealed that MDH2 and MYL3 could separate BMD patients and controls, whereas CA3 allowed for separation of DMD and BMD patients from each other in both plasma and serum.^43^ Two fragments of myofibrillar structural protein myomesin-3 (MYOM3) were found significantly elevated in *mdx* mice, GRMD dogs, and DMD patients compared to controls.^37^ Moreover, MYOM3 levels were decreased with age in DMD patients.^37^ Fibrinogen, S100 proteins, several coagulation and complement factors, and muscle-derived markers such as titin, myosin, and carbonic anhydrase I (CA1) were also identified to discriminate DMD patients and healthy controls.^44^

Proteome profiling of serum of *mdx* mice exposed a number of proteins that are altered, compared to control mice. Elevated proteins were mostly of muscle origin, whereas decreased proteins were mostly of extracellular origin.^45^ Proteomic analysis of serum of DMD patients validated the increased levels of most of the protein biomarkers identified in *mdx* mice. Importantly, some of the serum proteins in DMD patients show age-related changes similar to *mdx* mice.^45^^,^^46^ The establishment of clinical biomarkers for the DMD patients is equally important with the identification and establishment of biomarkers for the DMD animal models on which all of the therapeutic interventions are performed. Although DMD animal models share some characteristics of the disease, different biomarkers with the patients may be possibly identified.

Circulating serum metabolites associated with DMD revealed significant alterations in DMD patients.^47^ Interestingly, the ratio of two metabolites, creatine and creatinine, was significantly related to the progression of the disease in DMD patients.^47^^,^^48^ Two additional metabolites, creatinine and guanidinoacetic acid, showed intermediate levels in BMD patients compared to DMD patients and controls, suggesting that they can discriminate the two types of muscular dystrophies.^48^

Most research performed for the development of DMD protein biomarkers has been related to the identification of disease-specific biomarkers. Additional disease parameters, including the prognosis of disease progression and the response of the patients to therapeutic interventions, are currently gaining interest. It has been recently suggested that the serum levels of malate dehydrogenase 2 (MDH2) correlate with the stage of the disease and a patient’s response to treatment with corticosteroids.^49^ Interestingly, the serum levels of MDH2 are also associated with the risk of wheelchair dependency and pulmonary function, implying that MDH2 can potentially be used as a prognostic biomarker in DMD.^49^ Protein biomarkers were evaluated for their response to therapeutic interventions and their use as pharmacodynamic biomarkers. *mdx* mice treated with antisense oligonucleotides resulted in the restoration of MYOM3, ADAMTS5, and PGAM1 levels toward wild-type levels, implying that they could be potentially used as pharmacodynamic biomarkers.^37^^,^^46^ A proteome profile was also performed using serum samples from DMD patients with and without corticosteroid treatment.^50^ Corticosteroids are frequently administered to DMD patients to improve muscle strength and function. Significant alterations were determined in 22 proteins, including steroid hormones and inflammatory proteins. Of these, 17 proteins were confirmed in longitudinal studies involving pre-treated and post-treated DMD patients. The identified potential biomarkers were suggested to both the US Food and Drug Administration (FDA) and the European Medicines Agency (EMA) as exploratory biomarkers of efficacy and safety in the development of vamorolone, a potential replacement for corticosteroids.^50^ In a more recent study, proteomic analysis was performed in a group of young DMD patients before and after their treatment with glucocorticoids.^51^ This study showed that 17 serum proteins associated with DMD were normalized following glucocorticoid treatment, implying that they can be potentially used as pharmacodynamic biomarkers.^51^ In another study, the authors evaluated the response of *mdx* mice to deflazacort and omega-3, which are used in DMD patients to improve muscle strength and slow the progression of their disability. Treatment with these two anti-inflammatories altered the plasma levels of interleukins, providing evidence for their usefulness as pharmacodynamic biomarkers.^52^

The use of metabolomics for monitoring the disease was also examined, which showed promising results.^53^ In a 7-month longitudinal study performed in plasma of *mdx* and wild-type mice, a signature of 31 metabolites was identified to detect the disease progression beyond the known degeneration/regeneration phase.^53^ A number of the identified metabolites including dipeptides highly presented in muscle tissues were elevated in *mdx* plasma compared to controls, whereas other amino acids such as ornithine and glutamine were reduced.^53^

### Biomarkers for DM1 and DM2 Have Gained Attention

DM1 is the most common form of adult-onset muscular dystrophy. It is an autosomal dominant multisystemic disorder in which symptoms vary from mild to severe forms. DM1 is primarily characterized by progressive muscle weakness and wasting and may result in death due to pneumonia and respiratory insufficiency.^54^ DM1 is caused by a CTG repeat expansion on the 3′ untranslated region (3′ UTR) of the dystrophia myotonica protein kinase (*DMPK*) gene. A second type of myotonic dystrophy has been characterized, DM2, which shares similar symptoms with DM1, such as diabetes, muscular weakness, and cardiac failure.^54^ DM2 is caused by a CCTG repeat expansion on the CCHC-type zinc finger nucleic acid binding (*CNBP*) gene. The clinical onset of DM2 is usually in the third or fourth decade of the patient.

### Myotonic Dystrophy Biomarkers: The Emphasis on miRNAs

Recently, the development of biomarkers in DM1 has gained interest. A signature of nine deregulated miRNAs in plasma of DM1 patients was initially identified, suggesting their use as diagnostic biomarkers for DM1.^55^ Later, the levels of four myomiRs, that is, miR-1, miR-133a, miR-133b, and miR-206, were found elevated in the serum of DM1 patients, compared to controls, who showed minimal levels of these miRNAs in their serum.^56^ Importantly, the levels of the four myomiRs were significantly elevated in DM1 patients with progressive muscle wasting compared to patients with non-progressive muscle wasting (stable patients).^56^ In a larger cohort of DM1 patients, eight miRNAs (miR-140-3p, miR-454, miR-574, miR-1, miR-133a, miR-133b, miR-206, and miR-27b) were validated as plasma-based biomarkers for DM1.^57^ The validated miRNAs were also analyzed in the serum of DM1 patients, showing some variabilities, possibly due to different practical approaches such as the extraction kits or the variations in the molecular constituents of the starting material (plasma and serum).^58^ A deeper analysis of the disease stage and the clinical symptoms of all of the patients could also provide answers for the discrepancies of these miRNAs in blood. Seven of the miRNAs identified in DM1 patients (miR-1, miR-133a, miR-133b, miR-206, miR-140, miR-454, and miR-574) were also found deregulated in the plasma of a small group of DM2 patients.^57^ The identification of common biomarkers for different types of muscular dystrophies implies that the presence of some molecules in blood of the patients is possibly a consequence of muscle degradation and not a result of the disease and its cause.

In-depth investigation of the ontology of the four myomiRs in DM1 patients’ blood showed that these miRNAs are exclusively encapsulated within exosomes, and their exosomal levels correlate with muscle wasting observed in patients.^58^ The findings reported in DM1 differ from the findings reported in DMD, implying different pathogenic mechanisms that are involved in the two muscular dystrophies.^19^^,^^32^^,^^58^ Both DMD and DM1 primarily affect skeletal muscle tissue; however, different pathogenic mechanisms underlie these disorders. The absence of dystrophin protein in DMD patients damages muscle fibers and as a result they lose their function. Alternatively, DM1 is caused by a trinucleotide repeat expansion leading to nuclear RNA retention and abnormal alternative splicing. The differences in two muscular dystrophies possibly justify the different mechanisms by which skeletal muscle cells release muscle constituents within blood in these disorders.

### Circulating Biomarkers for Other Rare Muscular Dystrophies

Additional types of rare muscular dystrophies were investigated for the existence of circulating biomarkers. UCMD patients show a severe phenotype with generalized muscle weakness predominantly in the trunk and proximal limbs.^59^ The clinical symptoms appear soon after birth. MDC1A is a life-threatening disease characterized by neonatal onset of muscle weakness, severe hypotonia, progressive muscle weakness and wasting, and joint contractures.^60^ MDC1A is caused by mutations in the *LAMA2* gene encoding the α2 chain of laminin-211, a major constituent of the skeletal muscle basement membrane. FSHD is an adult-onset dominant inherited muscular dystrophy, characterized by a progressive weakness and atrophy of the skeletal muscles of the face, shoulder, arm, and abdominal muscles.^61^ Two forms of FSHD, FSHD1 and FSHD2, have been characterized, displaying an identical clinical phenotype but different genetic and epigenetic bases. LGMDs are a diverse group of rare diseases with many subtypes. The age of onset varies from childhood to adults of both sexes, and the clinical severity is very variable, ranging from mild to severe phenotypes. LGMDs usually cause weakness and wasting of the proximal muscles, including the muscles of the arms and legs.^62^ Regarding the development of biomarkers, work was performed on specific types of LGMDs, including LGMD2A, LGMD2B, LGMD2C, and LGMD2D. Due to the high heterogeneity and the increase in the characterization of new diseases in the family of LGMDs, a consortium was recently assigned to suggest an improved classification system for this large heterogeneous group of diseases.^3^ LGMDs were therefore proposed to be renamed based on the mode of inheritance (D, dominant; R, recessive), the order of discovery of the affected protein, and the affected protein.^3^ Based on the new nomenclature, LGMD2A, LGMD2B, LGMD2C, and LGMD2D were renamed as LGMD R1 calpain3-related, LGMD R2 dysferlin-related, LGMD R5 γ-sarcoglycan-related, and LGMD R3 α-sarcoglycan-related, respectively.^3^

### Rare Diseases with Limited Investigations

Despite the severity of UCMD, MDC1A, FSHD, and LGMDs and the necessity of development of clinical biomarkers to assess the progress of these diseases, very few studies have been performed. The levels of the four myomiRs, extensively studied in other muscular dystrophies, were investigated in UCMD, showing no difference in their levels compared to controls.^22^ On the contrary, miR-30c and miR-181a were elevated in UCMD compared to controls as observed in DMD and BMD, implying that they cannot be considered as disease-specific biomarkers.^28^ miR-30c and miR-181, however, can be considered as monitoring biomarkers of the muscle pathology, as these miRNAs are highly expressed in muscle and follow the same pattern in different muscular dystrophies. The levels of the myomiRs were also investigated in plasma of two animal models of MDC1A, dy^2J^/dy^2J^ and dy^3K^/dy^3K^ mice.^63^ myomiR levels were found significantly increased in both animal models compared to wild-type controls; however, no significant difference was observed between the two types of MDC1A mouse models.^63^ MDC1A is characterized by increased proteasomal activity, and proteasome inhibition using bortezomib was shown to partially improve muscle integrity in dy^3K^/dy^3K^ mice accompanied by a partial normalization of plasma levels of miR-1 and miR-133a.^63^ Administration of bortezomib, however, showed no effect on miR-206 plasma levels.^63^

The serum proteome was investigated in FSHD and LGMDs. High-throughput proteomics analysis identified a series of proteins that are altered in serum of FSHD patients when compared with controls.^64^^,^^65^ The identified proteins include skeletal muscle-specific proteins found in the serum of subjects with other muscular dystrophies such as DMD, proteins involved in protein folding and maintenance of aberrant cellular translation, inflammatory proteins, and proteins involved in cell adhesion, fusion, and migration that are related to pathogenic mechanisms involved in FSHD.^65^ Some of the identified biomarkers were suggested to be correlate with disease severity.^65^ MYOM3 fragments were identified to be elevated in serum of LGMD R3 α-sarcoglycan-related (LGMD2D) patients compared to controls; however, the levels of these fragments were lower in LGMD R3 α-sarcoglycan-related (LGMD2D) patients compared to DMD patients.^37^ The existence of circulating MYOM3 fragments was also investigated in four LGMD mouse models, that is, KO-calpain3 (model for LGMD R1 calpain3-related [LGMD2A]), KO-dysferlin (model for LGMD R2 dysferlin-related [LGMD2B]), KO-Sgcg (model for LGMD R5 γ-sarcoglycan-related [LGMD2C]), and KO-Sgca (model for LGMD R3 α-sarcoglycan-related [LGMD2D]), which showed interesting results.^37^ Following restoration of α-sarcoglycan expression in KO-Sgca mice the levels of MYOM3 fragments were restored, implying their use as pharmacodynamic biomarkers.^37^ In a parallel study, four proteins were reported to be significantly elevated in LGMD2B patients compared to controls and to correlate with the time needed to walk 10 m in patients.^42^ The identified proteins include sTnI, MYL3, FABP3, and the CKM protein that were also identified to be elevated in DMD and BMD patients.^42^ The creatine/creatinine ratio identified in DMD was also reported to be elevated in serum of LGMD R1 calpain3-related (LGMD2A) and LGMD R2 dysferlin-related (LGMD2B) patients.^48^

### Conclusions and Future Perspectives

During recent years, extensive research efforts have been made to identify clinical blood-based biomarkers for muscular dystrophies. A strong emphasis has been placed on DMD, although recently research focusing on the development of biomarkers for DM1 has come to the fore.^7^^,^^9^^,^^55^, ^56^, ^57^, ^58^ Sporadically, some possible biomarkers for UCMD, MDC1A, FSHD, and LGMDs have been reported (Table 1).^22^^,^^28^^,^^37^ Although very promising results have been reported, a lot of research is necessary to evaluate their use in clinical practice and define their use as monitoring and/or pharmacodynamics biomarkers.

**Table 1:** Circulating miRNAs and Proteins Identified as Potential Biomarkers for Muscular Dystrophies

Muscular Dystrophy	miRNAs	Proteins	Human	Animal Models	References
Duchenne muscular dystrophy (DMD)	miR-1 ↑		√	*mdx* mice/CXMD_J_ dogs/GRMD dogs	15, 16, 17, 18, 19, 20, 21, 22
	miR-133a ↑		√	*mdx* mice/CXMD_J_ dogs/GRMD dogs	15, 16, 17, 18, 19, 20, 21, 22
	miR-133b ↑		√	*mdx* mice/CXMD_J_ dogs/GRMD dogs	15, 16, 17, 18, 19, 20, 21, 22
	miR-206 ↑		√	*mdx* mice/CXMD_J_ dogs/GRMD dogs	15, 16, 17, 18, 19, 20, 21, 22
	miR-378 ↑			*mdx* mice	^19^
	miR-22 ↑			*mdx* mice	^19^^,^^26^
	miR-30a ↑			*mdx* mice	^19^
	miR-193b ↑			*mdx* mice	^19^
	miR-378 ↑			*mdx* mice	^19^
	miR-128 ↑			*mdx* mice	^26^
	miR-149 ↑			*mdx* mice	^26^
	miR-378b ↑			*mdx* mice	^26^
	miR-483 ↑		√	*mdx* mice	^26^
	miR-30c ↑		√		^27^^,^^28^
	miR-181a ↑		√		^27^^,^^28^
		MMP-9 ↑	√	*mdx* mice	38, 39, 40
		TIMP-1 ↑	√	*mdx* mice	^38^
		GDF-8 ↓	√		^39^
		FSTN ↑	√		^39^
		sTnI ↑	√	*mdx* mice/GRMD dogs	^42^
		MYL3 ↑	√	*mdx* mice/GRMD dogs	^42^^,^^43^
		FABP3 ↑	√	*mdx* mice/GRMD dogs	^42^
		CKM ↑	√	*mdx* mice/GRMD dogs	^42^
		fibronectin ↑	√		^41^
		CA3 ↑	√		^43^
		MDH2 ↑	√		^43^
		ETFA ↑	√		^43^
		MYOM3 ↑	√	*mdx* mice/GRMD dogs	^37^
		ADAMTS5 ↑	√	*mdx* mice	^46^
		PGAM1 ↑		*mdx* mice	^46^
		fibrinogen ↑	√		^44^
		S100 proteins ↑	√		^44^
		coagulation & complement factors ↑	√		^44^
		titin ↑	√		^44^
		myosin ↑	√		^44^
		CA1 ↑	√		^44^
		creatine ↑	√		^48^
		guanidinoacetic acid ↓	√		^48^
		creatine/creatinine ratio ↑	√		^47^^,^^48^
Becker muscular dystrophy (BMD)	miR-1 ↑		√		^18^^,^^22^
	miR-133a ↑		√		^18^^,^^22^
	miR-133b ↑		√		^18^^,^^22^
	miR-206 ↑		√		^18^^,^^22^
	miR-30c ↑		√		^27^^,^^28^
	miR-181a ↑		√		^27^^,^^28^
		sTnI ↑	√		^42^
		MYL3 ↑	√		^42^^,^^43^
		FABP3 ↑	√		^42^
		CKM ↑	√		^42^
		MDH2 ↑	√		^43^
		MMP-9 ↑	√		^39^
		FSTN ↑	√		^39^
Myotonic dystrophy type 1 (DM1)	miR-1 ↑		√		55, 56, 57, 58
	miR-133a ↑		√		55, 56, 57, 58
	miR-133b ↑		√		55, 56, 57, 58
	miR-206 ↑		√		55, 56, 57, 58
	miR-140-3p ↑		√		^55^^,^^57^^,^^58^
	miR-454 ↑		√		^55^^,^^57^^,^^58^
	miR-574 ↑		√		^55^^,^^57^^,^^58^
	miR-27b ↑/↓		√		^55^^,^^57^^,^^58^
Myotonic dystrophy type 2 (DM2)	miR-1 ↑		√		^57^
	miR-133a ↑		√		^57^
	miR-133b ↑		√		^57^
	miR-206 ↑		√		^57^
Ullrich congenital muscular dystrophy (UCMD)	miR-30c ↑		√		^28^
	miR-181a ↑		√		^28^
Congenital muscular dystrophy type 1A (MDC1A)	miR-1 ↑			dy^2J^/dy^2J^ mice/dy^3K^/dy^3K^ mice	^63^
	miR-133a ↑			dy^2J^/dy^2J^ mice/dy^3K^/dy^3K^ mice	^63^
	miR-133b ↑			dy^2J^/dy^2J^ mice/dy^3K^/dy^3K^ mice	^63^
	miR-206 ↑			dy^2J^/dy^2J^ mice/dy^3K^/dy^3K^ mice	^63^
Limb-girdle muscular dystrophy (LGMD)		sTnI ↑	LGMD2B		^42^
		MYL3 ↑	LGMD2B		^42^
		FABP3 ↑	LGMD2B		^42^
		CKM ↑	LGMD2B		^42^
		MYOM3 ↑	LGMD2	KO-Sgca mice/KO-calpain3 mice/KO-dysferlin mice/KO-Sgcg mice	^37^
		creatine/creatinine ratio ↑	LGMD2A LGMD2B		^48^

CXMD_J_, canine X-linked muscular dystrophy in Japan; GRMD, golden retriever muscular dystrophy; MMP-9, matrix metallopeptidase 9; TIMP-1, inhibitors of metalloproteinase-1; GDF-8, myostatin; FSTN, follistatin; sTnI, skeletal troponin I; MYL3, myosin light chain 3; FABP3, fatty acid binding protein 3; CKM, creatine kinase muscle type; CA3, carbonic anhydrase III; MDH2, mitochondrial malate dehydrogenase 2; ETFA, electron transfer flavoprotein A; MYOM3, myofibrillar structural protein myomesin-3; CA1, carbonic anhydrase I; 3′ UTR, 3′ untranslated region; DMPK, dystrophia myotonica protein kinase; CNBP, CCHC-type zinc finger nucleic acid binding; CAPN3, calpain-3; DYSF, dysferlin; SGCA, α-sarcoglycan; SGCG, γ-sarcoglycan.

It is essential to evaluate all of the potential biomarkers and the relative assay kits prior to their introduction to clinical practice. Evaluation studies and a fully analytical performance of the biomarker assays are necessary in order to provide the identified biomarkers to medical laboratories.^66^ Evaluation studies are required that will test various aspects of the biomarkers including their clinical purpose (e.g., example diagnosis, monitoring, or prognosis), the target condition and population, the specimen type, and the clinical interpretation of the results.^66^ A full analytical performance of the biomarkers is necessary that includes some crucial steps such as the required technology, result type, and reporting.^66^ Through the analytical performance the accuracy and trueness measurement, precision, limit of detection, analytical specificity, and other very important parameters will be evaluated in order to provide the identified biomarkers to medical laboratories.^66^

**Figure 2:**
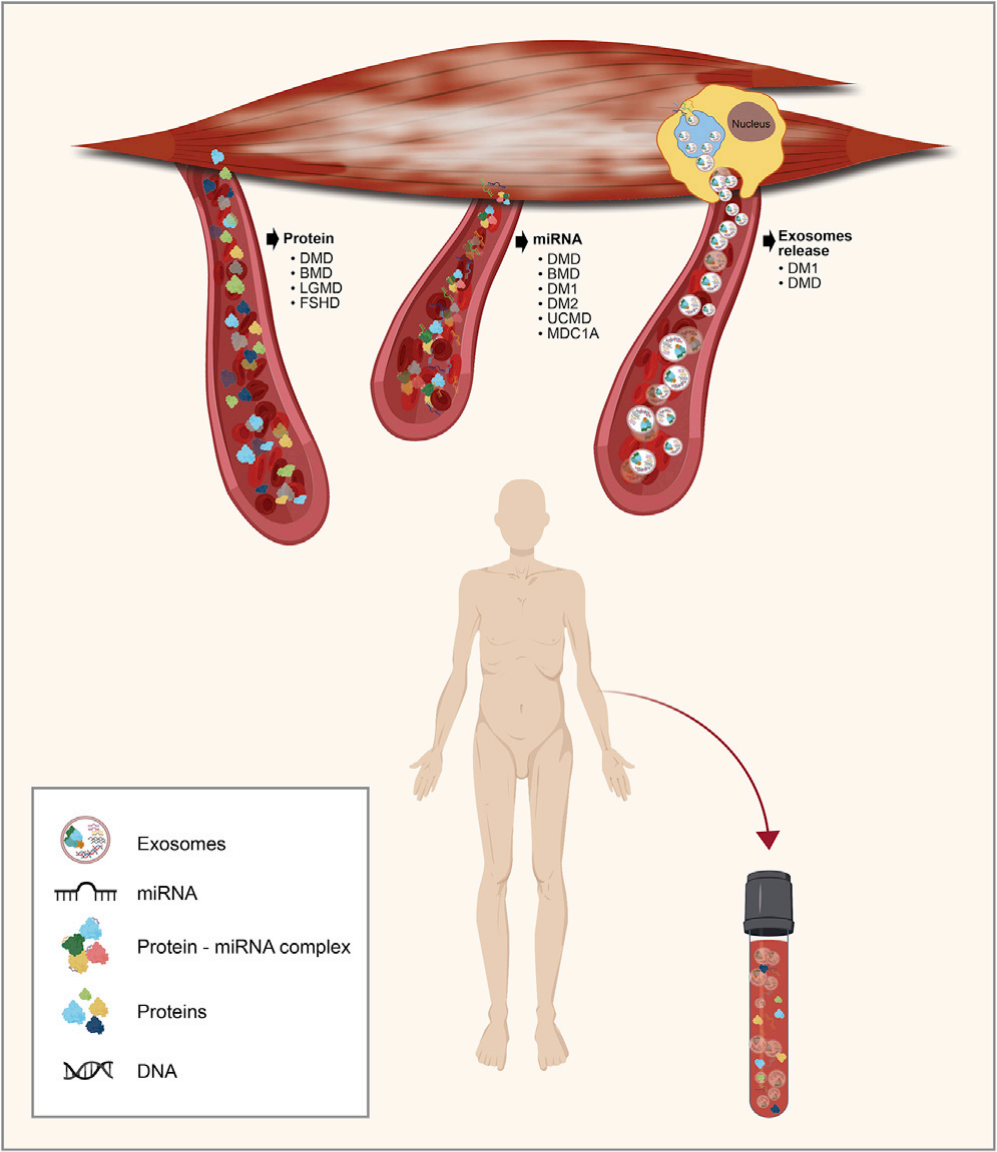
Circulating Biomarkers in Blood of Muscular Dystrophy Patients Muscular dystrophies are a heterogeneous group of inherited disorders sharing similar clinical and pathological characteristics. Their main common characteristic is the weakness, wasting, and degeneration of skeletal muscle. Currently, there is an increasing interest to develop reliable and measurable biomarkers for both the monitoring of the progress of muscular dystrophies (monitoring biomarkers) and the response to therapeutic approaches (pharmacodynamic biomarkers). Potential biomarkers have been identified for Duchenne muscular dystrophy (DMD), Becker muscular dystrophy (BMD), myotonic dystrophy types 1 and 2 (DM1 and DM2), Ullrich congenital muscular dystrophy (UCMD), congenital muscular dystrophy type 1A (MDC1A), facioscapulohumeral muscular dystrophy (FSHD), and limb-girdle muscular dystrophies (LGMDs). Several circulating proteins and miRNAs have been reported as potential biomarkers for the various muscular dystrophies. Furthermore, exosomal molecular cargo has been investigated and suggested as a source for biomarkers.

The identified biomarkers can serve two important roles in clinical practice. First, the disease-specific biomarkers that relate to the disease cause and pathogenic mechanism and can be useful for monitoring the response to disease-modifying therapies are important. Alternatively, disease non-specific biomarkers that are related to a specific symptom, such as muscle pathology, can serve as biomarkers that track the disease and/or as pharmacodynamic biomarkers for the improvement of the particular symptom. The identification of common biomarkers in different muscular dystrophies could lead to the establishment of biomarkers for the pathology of muscle tissue. This is of high importance for all muscular dystrophy patients. The development of biomarker panels, instead of a single biomarker, may prove a more robust readout tool for a better monitoring of the diseases. Furthermore, in multisystemic muscular disorders, such as DM1, a biomarker panel could be useful for evaluating all of the possible secondary complications that maybe develop in the patients. The identification of biomarker ontology is another important parameter that could possibly discriminate the different types of muscular dystrophies and clarify the precise clinical interpretation of the circulating biomarkers. For instance, exosomes can serve as a better source for circulating biomarkers, compared to the total population of proteins and/or miRNAs present in blood. In clinical practice, various laboratory procedures could be performed after collecting blood samples from patients. This could help to narrow down the screening material and the easier identification of the most appropriate biomarker (Figure 2).

## Conflicts of Interest

The authors declare no competing interests.
